# Alterations in the autonomic nerve activities of prenatal autism model mice treated with valproic acid at different developmental stages

**DOI:** 10.1038/s41598-020-74662-0

**Published:** 2020-10-20

**Authors:** Yoshiyuki Kasahara, Chihiro Yoshida, Kana Nakanishi, Miyabi Fukase, Arisa Suzuki, Yoshitaka Kimura

**Affiliations:** 1 Department of Maternal and Fetal Therapeutics, Tohoku University Graduate School of Medicine, Sendai, Japan; 2 Advanced Interdisciplinary Biomedical Engineering, Tohoku University Graduate School of Medicine, Sendai, Japan

**Keywords:** Diseases, Neurological disorders, Psychiatric disorders, Physiology, Cardiovascular biology, Neurophysiology, Disease model

## Abstract

Autism spectrum disorder (ASD) is characterized by impairment of social communication, repetitive behavior and restrictive interest. The risk of ASD is strongly associated with the prenatal period; for instance, the administration of valproic acid (VPA) to pregnant mothers increases risk of ASD in the child. Patients with ASD often exhibit an alteration in the autonomic nervous system. In this study, we assessed the autonomic nervous activity at each prenatal developmental stage of model mice of ASD treated with VPA, to clarify the relationship between timing of exposure and ASD symptoms. The assessment of the autonomic nervous activity was performed based on the analysis of electrocardiography data collected from fetal and adult mice. Interestingly, VPA model mouse fetuses exhibited a significantly lower activity of the sympathetic nervous system. In contrast, sympathetic nervous activity at P0 was significantly higher. In adult VPA model mice, the parasympathetic activity of female VPA mice was suppressed. Moreover, female VPA mice showed reduced the parasympathetic activity after exposure to restraint stress. These results suggest that the autonomic nervous activity of VPA model mice was altered from the fetal stage, and that the assessment of autonomic nervous activities at an early developmental stage could be useful for the understanding of ASD.

## Introduction

Autism spectrum disorder (ASD) is a developmental disorder characterized by disability of social communication, repetitive behavior, and restricted interests^1^. In this decade, developmental disorders such as ASD have been increasing^2^. In fact, the prevalence of ASD was 18.5 per 1,000 (one in 54) children aged 8 years in 2016, across 11 sites in the US^3^. The detailed mechanism underlying the pathogenesis of ASD remains unclear; hence, effective medications and an objective diagnosis have not been established for this condition^4^. Although no standard medication for ASD has been reported to date, early detection and appropriate care may improve its symptoms^5^.

The risk of developing ASD is strongly associated with the prenatal period. Maternal intake of certain chemical substances and antiepileptic drugs during pregnancy, genetic mutation in the fetus, and older parents are factors that increase the risk of developing ASD^6^. In particular, the consumption of valproic acid (VPA), which is an antiepileptic drug and mood stabilizer, by pregnant women triggers ASD and autism-like behavior in offspring of human and animal models, respectively^7–9^. Developmental Origins of Health and Disease (DOHaD) is a concept that entails that exposure to certain factors during the prenatal period may cause long-term side effects. This paradigm helps to understand how risk factors present during fetal development contribute to the triggering of several disorders. Psychiatric and developmental disorders have also been associated with DOHaD^10^. Prenatal maternal stress has been shown a risk factor that can causes changes in the social behavior of the offspring in humans and rodents during their adulthood^11,12^. In addition, prenatal maternal stress may induce cognitive disfunctions^13,14^, hyperactivity^15^, anxiety^16^, and depression-like behavior^17,18^.

The measurement of autonomic nervous system activation has been used to improve our understanding of the biological underpinnings of psychophysiological dysfunction^19–21^. The autonomic nervous system is considered to encompass three anatomical divisions: the sympathetic, parasympathetic, and the enteric nervous systems^22^. In particular, the sympathetic and parasympathetic nervous systems have opposing effects on various physiological activities. For example, the sympathetic nervous system accelerates the heart rate (HR), whereas the parasympathetic nervous system suppresses it. The activity of the autonomic nervous system can be accessed via the evaluation of HR variability (HRV) using electrocardiography (ECG)^23^. Power spectrum analysis of HRV can provide information on the balance of the sympathetic and parasympathetic activities that occur during numerous physiological and pathophysiological conditions. The power spectrum of HRV can be divided into two main domains, i.e., the low-frequency (LF) and high-frequency (HF) domains^24^. LF reflects the sympathetic and parasympathetic nerve activities, HF reflects the parasympathetic nerve activity, and the LF/HF ratio reflects the sympathetic nerve activity.

ECG analysis of children and adults diagnosed with ASD has revealed lower autonomic nervous activity compared with healthy individuals. This lower activity has been hinted to be associated with the pathology of ASD^25–27^. In addition, the sympathetic and parasympathetic interaction is associated with externalizing behavior problems in children with ASD^28^. In our previous studies, we developed techniques for measuring fetal ECG in humans^29–31^ and animals^23,32^, to evaluate the sympathetic nervous activity using ECG collected from fetuses and adult subjects.

As developmental and neuropsychiatric disorders, including ASD, can be associated with the concept of DOHaD, it is expected that early treatment and diagnosis will be possible by identifying the characteristics of the symptoms starting as early as the fetal stage. However, the elucidation of these mechanisms is, unfortunately, insufficient. Therefore, the evaluation of the autonomic nervous system at each developmental stage, starting from the fetal period, using ECG measurements may provide new insights into the pathophysiology of ASD. Furthermore, it may contribute to the establishment of diagnostic methods from the early stage of the developmental disorders. In this study, we assessed the development of autonomic nervous activity in both fetal and adult mice via ECG measurements in a mouse model of ASD generated via the administration of VPA during the fetal stage.

## Results

### Evaluation of sociability and social novelty in the VPA mouse model generated here (VPA mice)

**Figure 1:**
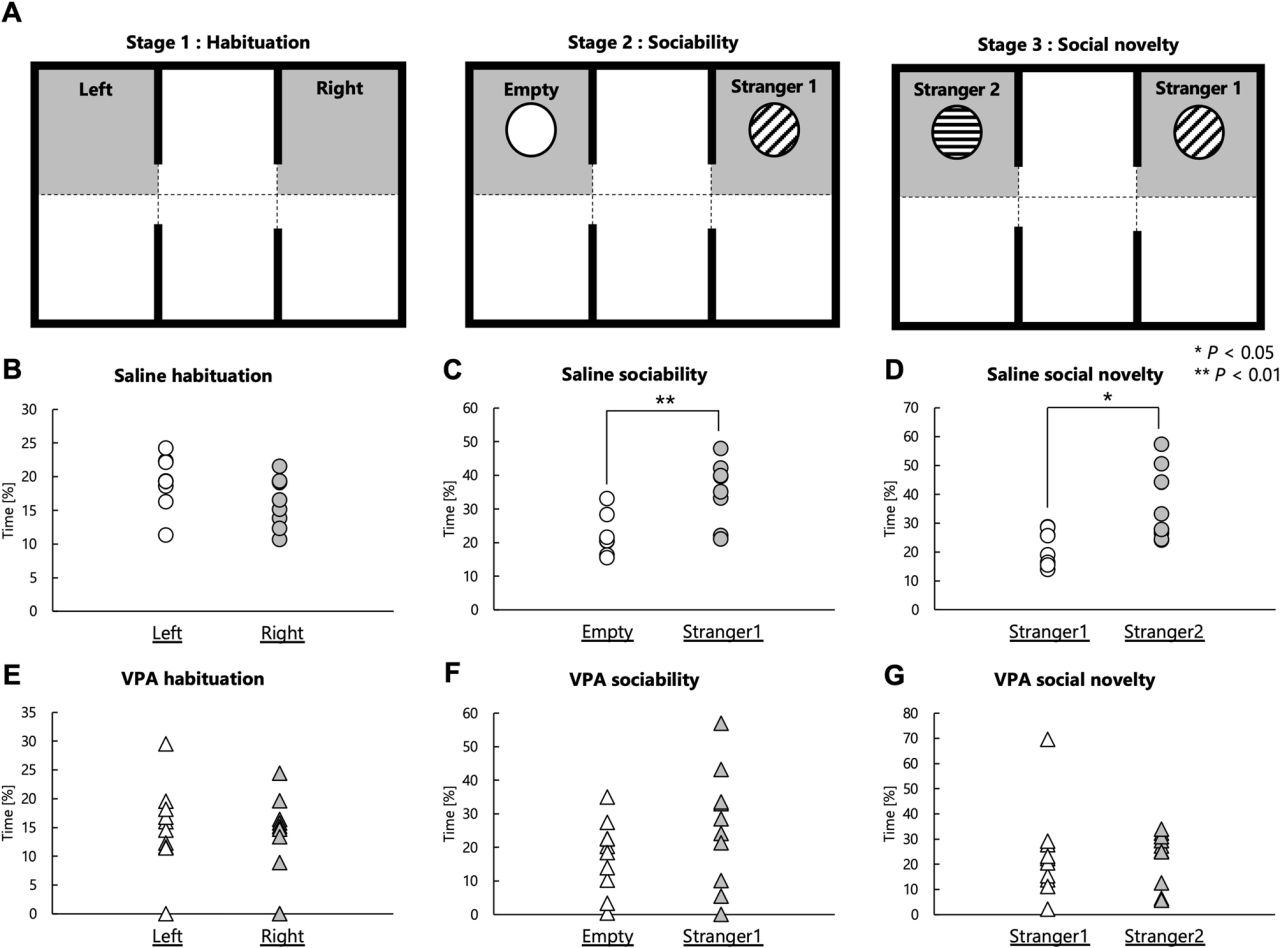
Evaluation of sociability and social novelty in the VPA mice used in this study. (**A**) Schematic illustration of the three-chambered test: first stage (habituation), second stage (sociability), and third stage (social novelty). The gray area was defined as the social-approach area. (**B**-**D**) Time spent by Sal mice in each of the social-approach areas during the habituation stage (**B**), sociability stage (**C**), and social novelty stage (**D**). (**E**–**G**) Time spent by VPA mice in each of the social-approach areas during the habituation stage (**E**), sociability stage (**F**), and social novelty stage (**G**). *P < 0.05, ** *P* < 0.01.

To confirm whether VPA mice were appropriate as a model of ASD under the experimental conditions of the present study, we performed the three-chambered social-approach test to evaluate the sociability and response to social novelty of VPA mice (Fig. 1A). During the habituation session, side preferences of mice were evaluated to exclude confounding effects. In the habituation session, both mouse groups, regardless of treatment, spent almost equal time roaming each side chamber (Fig. 1B,E). In the second session, a novel object (empty cage) along with a novel mouse (stranger 1) were introduced to the other two chambers to compare between the time spent with stranger 1 and the time spent with the empty cage. In the second session, male Sal mice spent more time with stranger 1 than the empty cage (*P* = 0.008; Fig. 1C). Conversely, male VPA mice spent a similar time in the area with stranger 1 and in the empty cage (*P* = 0.149) (Fig. 1F). In the third session, a second novel mouse (stranger 2) was placed in the cage that had been empty in the second session, to measure the time spent with familiar and unfamiliar mice. The results of the third session showed that male Sal mice spent more time with stranger 2 (unfamiliar) than stranger 1 (familiar) (*P* = 0.040; Fig. 1D). In contrast, male VPA mice did not distinguish the unfamiliar mouse from the familiar mouse (*P* = 0.927) (Fig. 1G). These results showed that the male VPA mice used in this study exhibited reduced social recognition.

### Autonomic nerve activities in fetal stages in autism model mice

**Figure 2:**
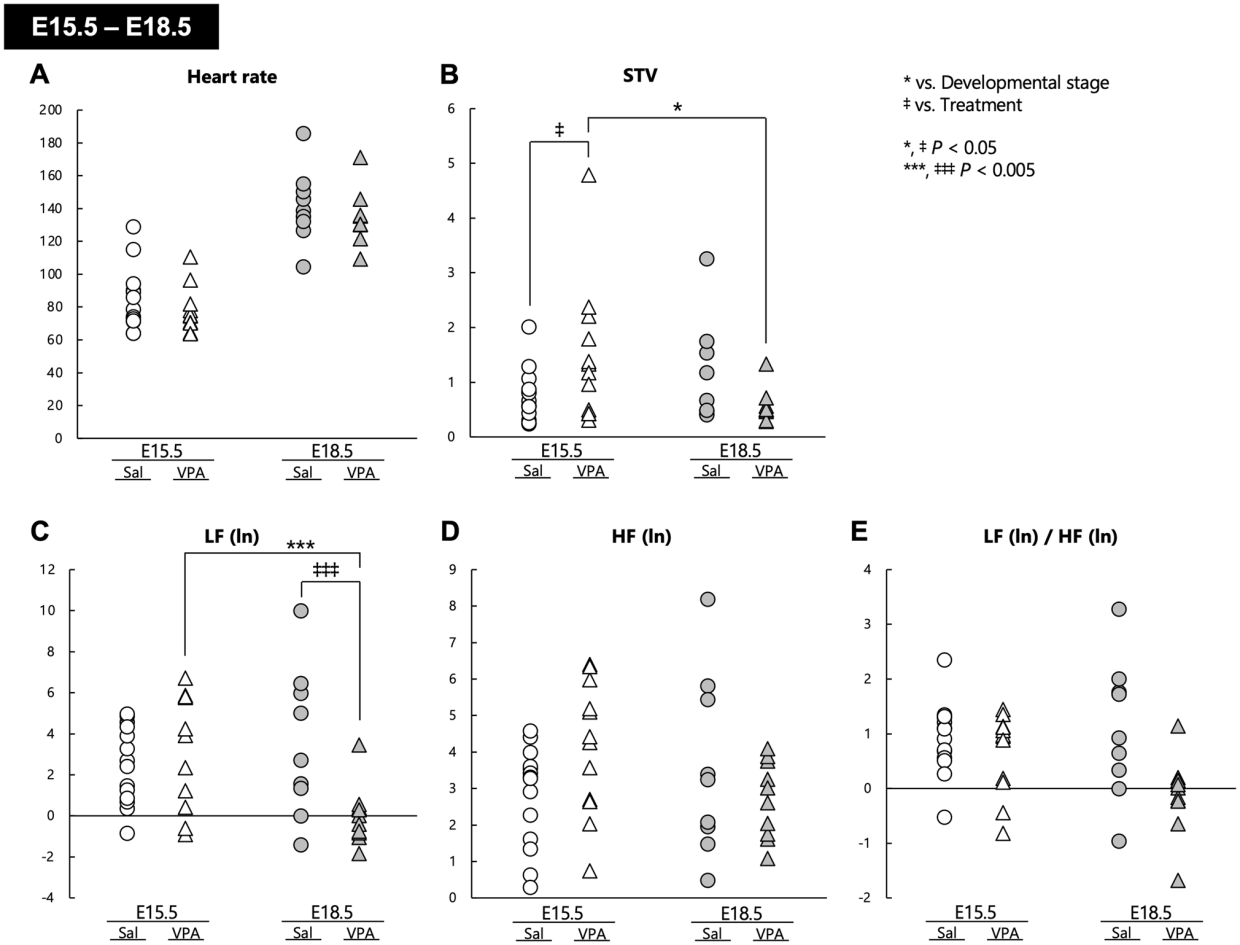
Autonomic nervous activities in fetal stages in both Sal and VPA mice. (**A**) Heart rate, (**B**) STV, (**C**) LF (ln), (**D**) HF (ln), and (**E**) LF (ln)/HF (ln) in the fetal stages of Sal and VPA mice at E15.5 and E18.5. * *P* < 0.05, *** *P* < 0.005 vs. developmental stage, ‡ *P* < 0.05, ‡‡‡ *P* < 0.005 vs. treatment.

In this study, we focused on the development of the autonomic nervous system in the fetal stage of a mouse model of autism established in-house; hence, we carried out fetal ECG measurements in these animals. Regarding HR, two-way analysis of variance (ANOVA) revealed the presence of a significant main effect of developmental stage (*F*[1, 41] = 114.35, *P* < 0.001), but no main effect of treatment (*F*[1, 41] = 1.404, *P* = 0.243) or the interaction of developmental stage with treatment (*F*[1, 41] = 0.006, *P* = 0.941) (Fig. 2A). Regarding the short-term variability (STV), there were no main effects of developmental stage (*F*[1, 41] = 1.088, *P* = 0.303) and treatment (*F*[1, 41] = 0.121, *P* = 0.730); however, a significant interaction of developmental stage with treatment (*F*[1, 41] = 6.603, *P* = 0.014) was observed. Post hoc comparisons demonstrated that the STV at E18.5 was significantly lower than that at E15.5 in VPA mice (*P* = 0.015), and that the STV of VPA mice at E15.5 was significantly higher than that of Sal mice (*P* = 0.030) (Fig. 2B). There were no main effects of the developmental stage on the LF (ln) (*F*[1, 41] = 2.473, *P* = 0.124), HF (ln) (*F*[1, 41] = 0.395, *P* = 0.533), and LF (ln)/HF (ln) ratio (*F*[1, 41] = 1.585, *P* = 0.215). Although no main effect of treatment was observed on the LF (ln) (*F*[1, 41] = 2.977, *P* = 0.092) and HF (ln) (*F*[1, 41] = 0.221, *P* = 0.641), a significant main effect of treatment on LF (ln)/HF (ln) was detected (*F*[1, 41] = 8.237, *P* = 0.006). The interaction of developmental stage with treatment on the LF (ln) (*F*[1, 41] = 8.622, *P* = 0.005) and HF (ln) (*F*[1, 41] = 4.437, *P* = 0.041) was significant, whereas no interaction of developmental stage with treatment on the LF (ln)/HF (ln) was observed (*F*[1, 41] = 3.638, *P* = 0.064). Post hoc comparisons showed that the LF (ln) of VPA mice was significantly decreased at E18.5 compared with at E15.5 (*P* = 0.003). In addition, the LF (ln) of VPA mice at E18.5 was significantly lower than that of Sal mice (*P* = 0.004) (Fig. 2C–E).

**Figure 3:**
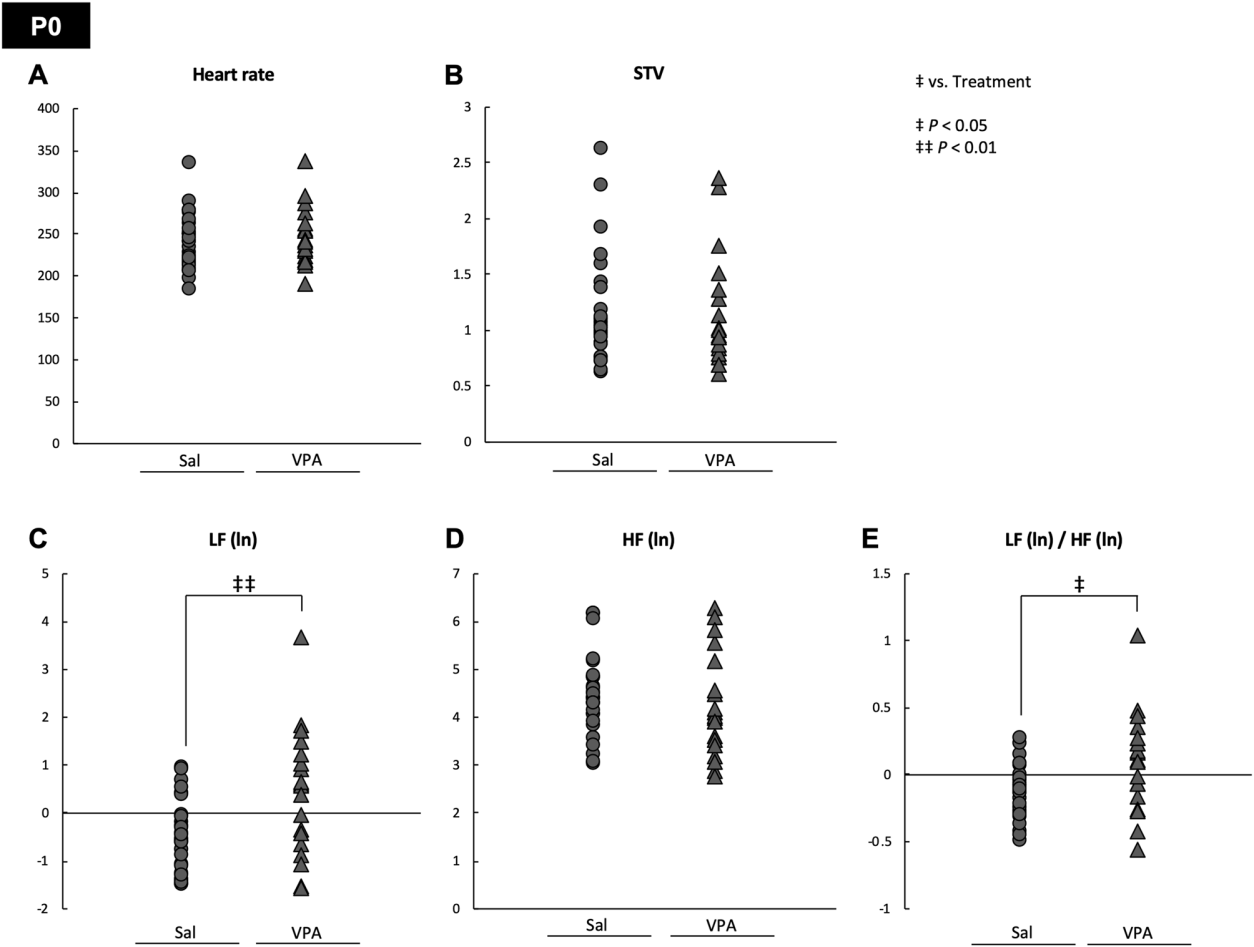
The neonatal sympathetic activity was higher in VPA mice. The (**A**) heart rate, (**B**) STV, (**C**) LF (ln), (**D**) HF (ln), and (**E**) LF (ln)/HF (ln) at P0 were measured in Sal and VPA mice at P0. The LF (ln) and LF (ln)/HF (ln) were significantly higher in VPA mice. ‡ *P* < 0.05, ‡‡ *P* < 0.01 vs. treatment.

### Neonatal sympathetic activity was higher in VPA infants

We assessed the ECG results in VPA and Sal mice at P0. One-way ANOVA indicated that there were no main effects of treatment on HR (*F*[1, 45] = 0.355, *P* = 0.554), STV (*F*[1, 45] = 0.007, *P* = 0.932), and HF (ln) (*F*[1, 45] = 0.428, *P* = 0.516), whereas significant main effects of treatment on LF (ln) (*F*[1, 45] = 8.725, *P* = 0.005) and LF (ln)/HF (ln) (*F*[1, 45] = 7.112, *P* = 0.011) were detected (Fig. 3A–E). The latter results are the opposite to those obtained for E18.5 of VPA mice (Fig. 2A–E).

### The alterations of autonomic nervous activities observed in the fetal stage of VPA mice were partially ameliorated over time in the postnatal period

**Figure 4:**
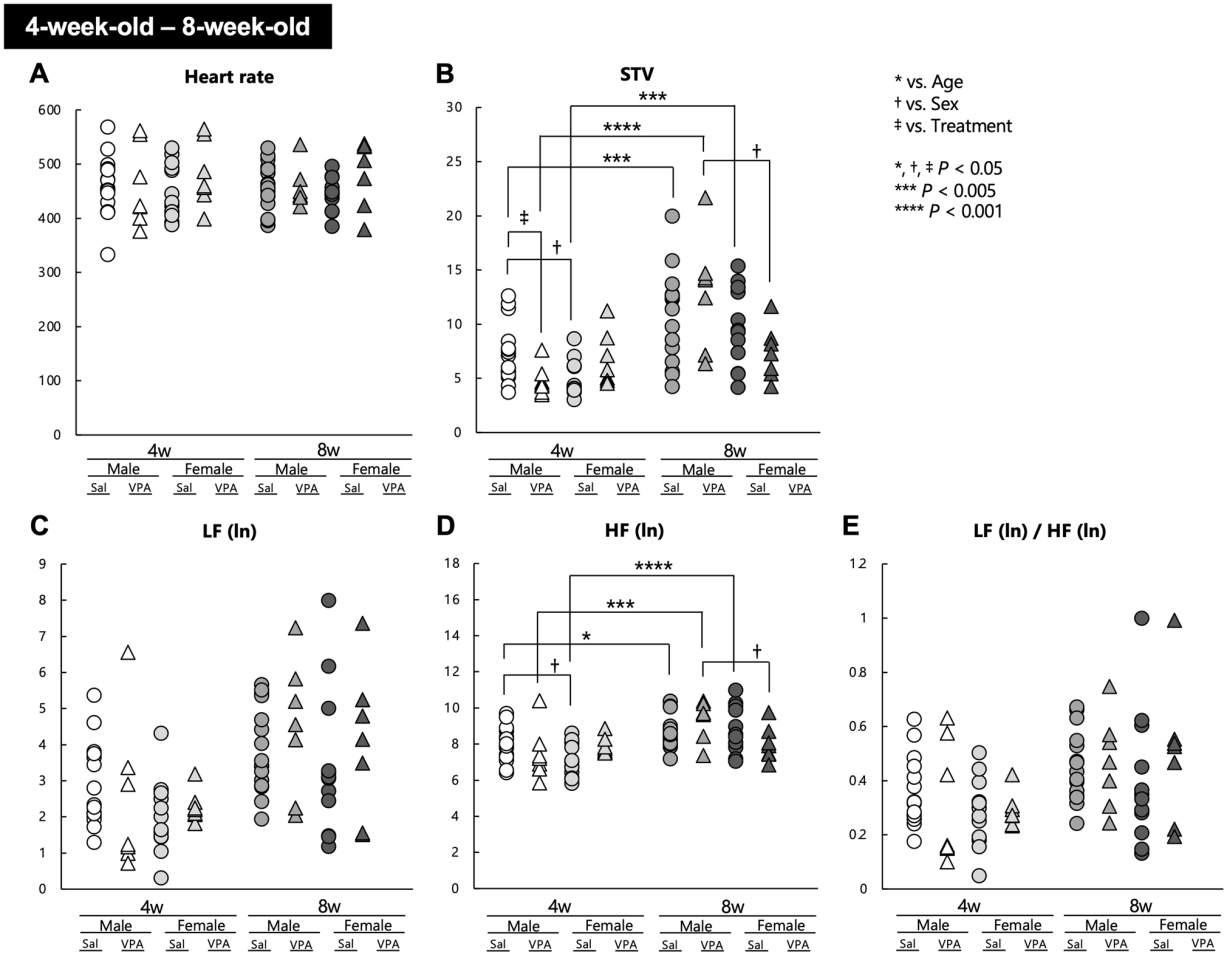
Heart rate and its variability in 4- to 8-week-old Sal and VPA mice. The (**A**) heart rate, (**B**) STV, (**C**) LF (ln), (**D**) HF (ln), and (**E**) LF (ln)/HF (ln) of 4- to 8-week-old Sal and VPA mice were plotted. * *P* < 0.05, *** *P* < 0.005, **** *P* < 0.001 vs. age. † *P* < 0.05 vs. sex, ‡ *P* < 0.05 vs. treatment.

We performed ECG measurements in VPA and Sal mice at 4 and 8 weeks of age. Three-way repeated measures ANOVA revealed that there were no main effects of age (*F*[1, 38] = 0.011, *P* = 0.915), sex (*F*[1, 38] = 0.284, *P* = 0.597), and treatment (*F*[1, 38] = 1.643, *P* = 0.208) on HR. Moreover, no significant age × sex × treatment interaction was observed regarding HR (*F*[1, 38] = 0.117, *P* = 0.734). No significant simple interaction effects were found for age × sex (*F*[1, 38] = 0.010, *P* = 0.920), age × treatment (*F*[1, 38] = 0.029, *P* = 0.886), and sex × treatment (*F*[1, 38] = 1.550, *P* = 0.221) (Fig. 4A). Regarding the STV, there were significant main effects of age (*F*[1, 38] = 36.324, *P* < 0.001) and sex (*F*[1, 38 = 4.805], *P* = 0.035), but no main effect of treatment (*F*[1, 38] = 0.034, *P* = 0.855). A significant age × sex × treatment interaction was observed (*F*[1, 38] = 9.810, *P* = 0.003). No significant simple interaction effects were found for age × treatment (*F*[1, 38] = 0.036, *P* = 0.850) and sex × treatment (*F*[1, 38] = 0.010, *P* = 0.923), whereas a significant simple interaction effect was observed for age × sex (*F*[1, 38] = 5.141, *P* = 0.029). Post hoc comparisons revealed that male Sal mice at 4 weeks had a higher STV than did female Sal mice (*P* = 0.010), whereas male VPA mice at 8 weeks had a higher STV than did female VPA mice (*P* = 0.012). In addition, the STV of male VPA mice at 4 weeks was lower comparable to that of male Sal mice (*P* = 0.022), whereas at 8 weeks, there was no significant difference between treatments in male mice (*P* = 0.221). In female mice, no alterations were observed both at 4 (*P* = 0.089) and 8 (*P* = 0.237) weeks between treatments. Conversely, the STV of male Sal mice, male VPA mice, and female Sal mice was significantly increased at 8 weeks vs. 4 weeks of age (*P* = 0.002, *P* < 0.001 and *P* < 0.001, respectively), whereas the STV of female VPA mice did not vary according to age (*P* = 0.724) (Fig. 4B). Regarding the LF (ln), there was a significant main effect of age (*F*[1, 38] = 20.196, *P* < 0.001), whereas no significant effects were demonstrated for sex (*F*[1, 38] = 1.731, *P* = 0.196) and treatment (*F*[1, 38] = 0.623, *P* = 0.435). No significant age × sex × treatment interaction was observed (*F*[1, 38] = 0.262, *P* = 0.611). No significant simple interaction effects were found for age × sex (*F*[1, 38] = 0.003, *P* = 0.955), age × treatment (*F*[1, 38] = 0.893, *P* = 0.351), and sex × treatment (*F*[1, 38] = 0.239, *P* = 0.627) (Fig. 4C). Regarding the HF (ln), there was a significant main effect of age (*F*[1, 38] = 24.841, *P* < 0.001), but no significant effects were observed for sex (*F*[1, 38] = 2.655, *P* = 0.112) and treatment (*F*[1, 38] = 0.165, *P* = 0.687). A significant age × sex × treatment interaction was observed (*F*[1, 38] = 9.951, *P* = 0.003). No significant simple interaction effects were found for age × sex (*F*[1, 38] = 0.782, *P* = 0.382), age × treatment (*F*[1, 38] = 0.241, *P* = 0.626), and sex × treatment (*F*[1, 38] = 0.013, *P* = 0.910). Post hoc comparisons demonstrated that male Sal mice at 4 weeks had a higher HF (ln) than did female Sal mice (*P* = 0.024), and that male VPA mice at 8 weeks had a higher HF (ln) than did female VPA mice (*P* = 0.023). The HF(ln) values of male Sal mice, male VPA mice, and female Sal mice were significantly increased at 8 weeks vs. 4 weeks of age (*P* = 0.042, *P* = 0.001, and *P* < 0.001, respectively), whereas the HF(ln) of female VPA mice remained unchanged with aging (*P* = 0.845) (Fig. 4D). Regarding the LF (ln)/HF (ln) ratio, there was a significant main effect of age (*F*[1, 38] = 12.418, *P* = 0.001), but no significant effects were demonstrated for sex (*F*[1, 38] = 0.519, *P* = 0.476) and treatment (*F*[1, 38] = 0.369, *P* = 0.547). No significant age × sex × treatment interaction was observed (*F*[1, 38] = 0.035, *P* = 0.854). No significant simple interaction effects were found for age × sex (*F*[1, 38] = 0.223, *P* = 0.640), age × treatment (*F*[1, 38] = 0.863, *P* = 0.359), and sex × treatment (*F*[1, 38] = 0.679, *P* = 0.415) (Fig. 4E).

The results obtained at 4 and 8 weeks of age in VPA mice suggest that the disturbances in autonomic nervous activities, which were notable in the fetal and neonatal stages of VPA mice, were relieved over time in the postnatal period, especially regarding the sympathetic nervous activity; however, abnormal regulation of the parasympathetic nervous system seemed to persist into the adult stage of development.

### Female VPA mice show lower parasympathetic activity under the stress condition

**Figure 5:**
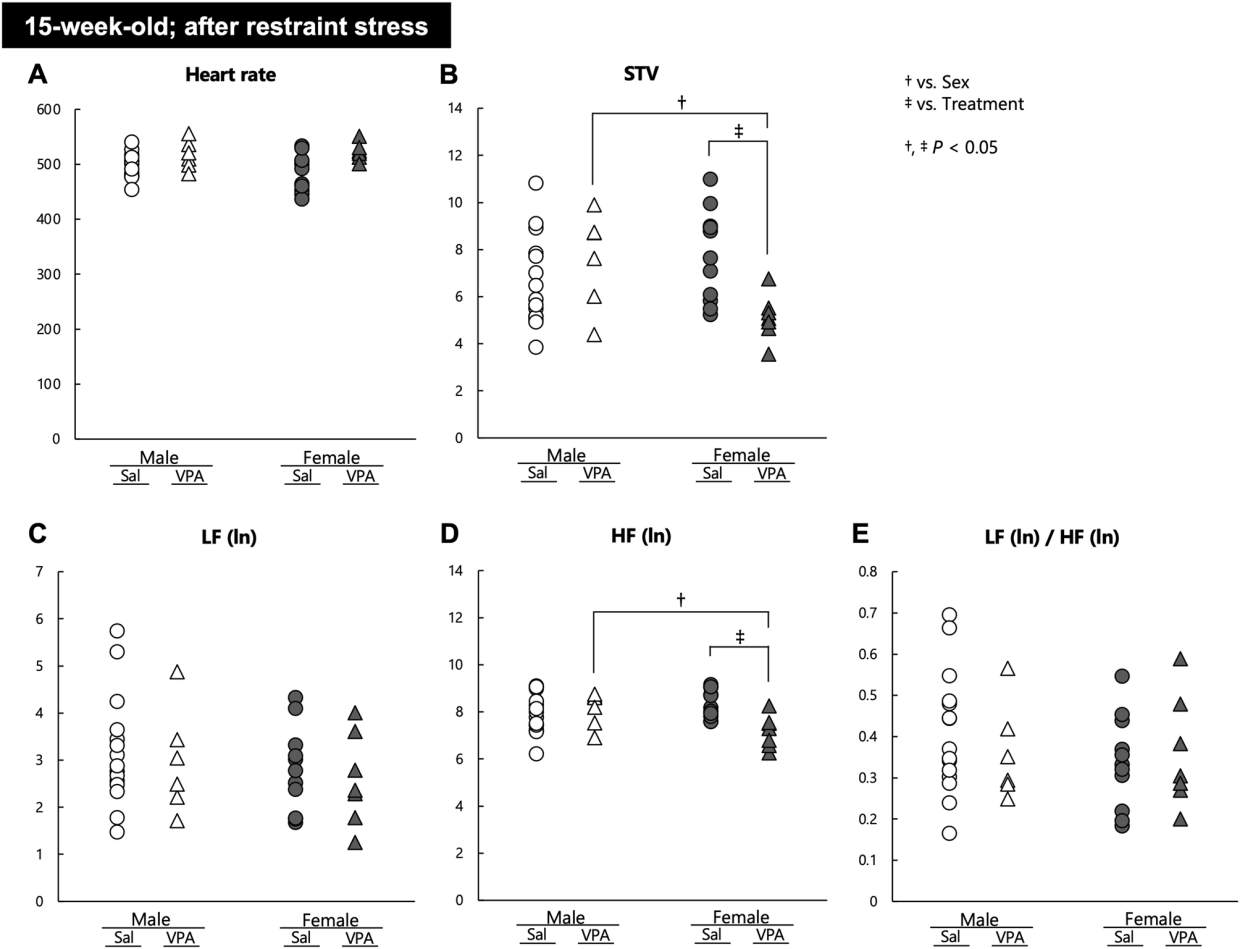
Autonomic nervous activities after exposure to a restraint stress in 15-week-old Sal and VPA mice. The (**A**) heart rate, (**B**) STV, (**C**) LF (ln), (**D**) HF (ln), and (**E**) LF (ln)/HF (ln) of 15-week-old Sal and VPA mice were evaluated after restraint stress in mice of both sexes. † *P* < 0.05 vs. sex, ‡ *P* < 0.05 vs. treatment.

Finally, we evaluated the autonomic responses of 15-weeks-old VPA and Sal mice after exposure to a 60 min restraint stress. Regarding HR, two-way ANOVA revealed that there was a significant main effect of treatment (*F*[1, 35] = 12.251, *P* = 0.001), but no main effect of sex (*F*[1, 35] = 0.088, *P* = 0.769) or interaction of sex with treatment (*F*[1, 35] = 0.957, *P* = 0.335) (Fig. 5A). Regarding STV, there were no main effects of sex (*F*[1, 35] = 1.129, *P* = 0.295) and treatment (*F*[1, 35] = 1.705, *P* = 0.200), whereas a significant interaction of sex with treatment (*F*[1, 35] = 8.260, *P* = 0.007) was observed. Post hoc comparisons demonstrated that the STV of male VPA mice was significantly higher than that of female VPA mice (*P* = 0.021), and that the STV of female VPA mice was significantly lower than that of female Sal mice (*P* = 0.006) (Fig. 5B). Regarding LF(ln), there were no main effects of sex (*F*[1, 35] = 1.109, *P* = 0.299), treatment (*F*[1, 35] = 0.327, *P* = 0.571), and interaction of sex with treatment (*F*[1, 35] = 0.000, *P* = 0.991) (Fig. 5C). In turn, regarding HF(ln), there were no main effects of sex (*F*[1, 35] = 0.823, *P* = 0.370) and treatment (*F*[1, 35] = 3.265, *P* = 0.079), whereas a significant interaction of sex with treatment (*F*[1, 35] = 8.135, *P* = 0.007) was observed. Post hoc comparisons showed that the HF(ln) of male VPA mice was significantly higher than that of female VPA mice (*P* = 0.027), and that the HF(ln) of female VPA mice was significantly lower than that of female Sal mice (*P* = 0.002) (Fig. 5D). Finally, regarding the LF(ln)/HF(ln) ratio, there were no main effects of sex (*F*[1, 35] = 0.640, *P* = 0.429), treatment (*F*[1, 35] = 0.080, *P* = 0.778), and interaction of sex with treatment (*F*[1, 35] = 0.577, *P* = 0.453) (Fig. 5E).

The results of the restraint stress experiment suggested that female VPA mice were more prone to restraint stress because they experienced abnormal alterations in the parasympathetic regulation.

## Discussion

It was previously shown that prenatal exposure to VPA results in reduced sociability in male mice^33,34^. Therefore, we examined the sociability of the male VPA mice generated in this study and tested their validity as an ASD model. Male Sal mice exhibited sociability and social novelty, whereas male VPA mice had impaired sociability and social novelty (Fig. 1B-G). Thus, the male VPA mice used in our study exhibited an autism-like behavior.

Here, we performed ECG measurements in ASD model mice at fetal stages (E15.5 and E18.5) and after birth (P0 and 4, 8 and 15 weeks of age). The ECG at 15 weeks of age was performed after exposure to stress. Different features were calculated from the ECG data to identify autonomic nervous system regulation patterns that could assist in the diagnosis of ASD from early developmental stages.

Fetal ECG data collected at E15.5 and E18.5 indicated that the sympathetic nerve activity of VPA mice was significantly decreased regardless of developmental stage, because a significant main effect of treatment on the LF (ln)/HF (ln) ratio was observed in VPA mice. Although the HF (ln) of VPA mice was not affected by developmental stage and treatment, the STV of VPA mice was reduced at E18.5 compared with E15.5. Moreover, the LF (ln) of VPA mice was decreased at E18.5 compared with E15.5. However, the STV, LF (ln), and HF (ln) were not changed in Sal mice between E15.5 and E18.5. These results suggest that both the sympathetic and parasympathetic nervous activity decreased in VPA mice as fetal development progressed. The STV of VPA mice was higher than that of Sal mice at E15.5, whereas the HF (ln) of VPA mice was not changed compared with that of Sal mice at E15.5. Because the STV is an indicator of parasympathetic activity, the higher STV observed in VPA vs. Sal mice at E15.5 suggests increased activity of the parasympathetic nervous system; however, the discrepancy between the STV and HF (ln) observed at E15.5 might reflect changes other than parasympathetic function at earlier stages of fetal development. Moreover, the LF (ln) of VPA mice was significantly lower than that of Sal mice at E18.5 (Fig. 2A–E). Lower LF (ln) and LF (ln)/HF (ln) values indicate decreased sympathetic nerve function. Furthermore, decreased sympathetic nerve activity was observed in the VPA group, which suggests that fetal sympathetic nerve function is associated with the abnormalities observed in the ASD model during fetal development. Therefore, the assessment of the sympathetic nerve activity may be useful for the early diagnosis of ASD.

It has been reported that the expression of the *HAND2* gene, which encodes a transcription factor and is required for sympathetic neuron survival^35^, was decreased in neurons derived from human Chr. 15q11.2–q13.1 duplicated ASD stem cells^35^. Hence, our results of lower activities of the sympathetic nervous system in the fetal stage of the ASD model mice could explain the impaired sympathetic survival and/or development detected in ASD.

Based on several previous reports (see the reviews^7,36^), we injected pregnant female mice with VPA at E12.5 to create a model of ASD. It has been suggested that neural crest cell (NCC) differentiation toward sympathetic and parasympathetic neurons depends on the expression of their neurotransmitters and the synthesis of enzymes and transcription factors^37^. Around E12.5, migrating NCCs play a role in the development of cardiac innervation and conduction^37^. Although VPA has multiple putative actions, including an increase in γ-aminobutyric acid (GABA) levels, a decrease in aspartate levels, and the blockade of sodium, calcium, and potassium voltage-gated channels, it has been suggested that histone deacetylase (HDAC) inhibition underlies the causal relationship between VPA and ASD^9,38^. As HDAC enzymes regulate the conformation of chromatin and affect gene transcription, exposure of the fetuses to VPA around E12.5 might have affected the development of sympathetic nerves of the heart via changes in gene expression. It is difficult to explain why the sympathetic activity was altered in VPA mice. However, because a significant main effect of treatment on LF (ln)/HF (ln) was observed, impairment of sympathetic differentiation may have occurred up to E15.5. We speculate that the delay in sympathetic development became more apparent at E18.5 vs. E15.5. Genes such as *BMP*, *MASH1*, *PHOX2B*, *GATA3* and *HAND2* are important for NCC differentiation into sympathetic neurons^37^; therefore, these genes would be involved in the changes in sympathetic activity caused by prenatal VPA administration. Thus, the prenatal administration of VPA changes the expression level of various molecules, which might have affected the development and/or maturation of the autonomic nervous system. The prenatal administration of an HDAC inhibitor caused changes in the expression of autism-related genes, including *Shank 3*, which is associated to a delay in neuronal maturation^39^. In addition, exposure to VPA during pregnancy induces a reduction of the phosphatase and tensin homolog (PTEN) levels^40^, and *PTEN* knockout mice exhibit autism-like behaviors^41,42^. As described above, it can be inferred that prenatal VPA caused autism-like phenotypes through various different effects and contributed to changes in autonomic function.

Interestingly, at the P0 neonatal stage, VPA mice showed a significantly higher LF (ln) and LF (ln)/HF (ln) compared with Sal mice (Fig. 3A–E). Hence, the LF (ln) and LF (ln)/HF (ln) values were significantly lower during the fetal stage in VPA mice compared with Sal mice; nevertheless, the results obtained at P0 are difficult to interpret at first glance. One possibility is that VPA mice might have had an immature regulation of the sympathetic nervous system. The P0 neonatal mice had just undergone labor, which is a very strong stressor. Because we performed ECG measurements in neonatal mice several hours after parturition, we speculate that the sympathetic nerve activity evoked by parturition in Sal mice had recovered. In contrast, the sympathetic nerve activity of VPA mice had remained active because of immature regulation. Sheep studies have shown that neonatal blood noradrenaline levels are increased 1 h after parturition; however, 6 h after parturition, blood noradrenaline levels were decreased^43^. Conversely, some articles have indicated that high levels of LF persisted for several hours after parturition in humans^44^ and sheep^43^. The latter reports are inconsistent with the results of this study. As no previous report has examined the postpartum sympathetic nerve activity in mice in detail, it is necessary to study mice by focusing on postpartum sympathetic function in the future, to understand properly the results of the present study. Moreover, delivery radically switches the infant's circulation, breathing and physiology^45,46^. The hyperactivation of the sympathetic nervous system observed in VPA mice at P0 may reflect an impaired physiological switch from fetal to neonatal physiology, which might be central to the neuropathophysiology of VPA mice.

The results of the ECG data analysis performed at 4 and 8 weeks of age revealed the presence of a significant age × sex × treatment interaction regarding the STV and HF (ln), whereas only a main effect of age was observed on the LF (ln) and LF (ln)/HF (ln) (Fig. 4A–E). The STV and HF are indicators of the activity of the parasympathetic nervous system^24,47^; thus, we thought that the function of parasympathetic control was affected to a greater extent at these developmental stages in the present study. Post hoc comparisons revealed that, although the HF (ln) was not significantly different, male VPA mice had a significantly lower STV than did male Sal mice at 4 weeks of age. Therefore, VPA treatment during pregnancy may affect the parasympathetic nerve activities of male mice at 4 weeks of age. Interestingly, at this age, both the STV and HF (ln) of Sal mice were higher in male mice compared with female mice, whereas no sex differences were observed in VPA mice. Conversely, at 8 weeks of age, both the STV and HF (ln) of male VPA mice were higher compared with female VPA mice. The STV and HF (ln) were not different between male Sal mice and female Sal mice at 8 weeks of age. These results suggest that parasympathetic maturation is delayed in VPA mice; hence, the appearance of sex differences may have been shifted to a later stage of development compared with Sal mice. The STV and HF (ln) of male Sal, male VPA, and female Sal mice were significantly increased from 4 to 8 weeks of age, whereas these of female VPA mice remained unchanged, suggesting the strong suppression of parasympathetic nervous development in female VPA mice. Several reports revealed that the assessment of the sympathetic and parasympathetic activity using HRV in children with ASD yielded inconsistent results^25–27,48^. As ASD is a heterogeneous disease^1^, it can be inferred that such diversity occurred because of the characteristics of the population and the environmental factors that were involved in the studies. Because deficits in synaptic pruning by microglia may underlie the pathogenesis of ASD^49,50^ and microglial changes have been reported in VPA mice^51,52^, it is possible that synaptic pruning in the autonomic nervous system at the appropriate time is impaired in VPA mice. The enteric nervous system, which is an autonomic nervous system, contributes strongly to ASD symptoms^53^; therefore, the assessment of the enteric nervous system in VPA mice may be important to understand to our results. Moreover, ASD symptoms and the function of the autonomic nervous system vary according to sex^54,55^. Phenotypic sex differences have also been reported for VPA mice: sociability in VPA mice is strongly impaired in males, whereas social impairment is limited in females^33,34^. The relationship between sex and the symptoms caused by prenatal VPA administration is very important in our study, and there is a need to understand these sex differences better; for example, the relationship between the autonomic nervous system and sex hormones. In our study, a significant main effect of sex was observed in STV, whereas the main effect of treatment on STV was not significant. Therefore, it was possible that the effect of sex could be greater than the effect of treatment, at least in this study. More detailed and extensive research using various animal models of ASD in both sexes will be needed to elucidate the relationship between ASD and the autonomic nervous system.

Stress is known to worsen ASD symptoms^56^. Therefore, we assessed autonomic nervous activities in VPA and Sal mice after exposure to acute physical stress. A significant sex × treatment interaction was observed for both the STV and HF (ln) after exposure to acute stress, whereas the LF (ln) and LF (ln)/HF (ln) were not significantly changed. Female VPA mice had lower STV and HF (ln) than did male VPA mice, as well as lower STV and HF (ln) compared with female Sal mice. These results indicate that parasympathetic nervous activities after stress stimuli were suppressed in female VPA mice. In this condition, a significant main effect of treatment was detected in the HR analysis, i.e., a higher HR was observed in VPA mice compared with Sal mice (Fig. 5A–E). Stress stimuli evoke the upregulation of sympathetic nervous activity, which increases the HR. Subsequently, the parasympathetic nervous activity increases, causing the HR to decrease, for calming down and returning to the normal condition. We performed ECG measurements in mice after 60 min of restraint stress and 15 min of restoration. Thus, our result suggests that female VPA mice failed to recover from stress because of the absence of upregulation of the parasympathetic nervous activity. Because female sex hormones affect autonomic control^57^, sex hormones may be responsible for the stress vulnerability observed in the female VPA mice in this study. Long-term mental stress may worsen ASD symptoms more than physical stress. Although there were no significant differences in the VPA male mouse group after exposure to physical stress, male mice might be more vulnerable to other types of stress, such as mental stress. The vulnerability of mice to different types of stress needs to be investigated in future studies.

This study has several limitations. First, the effect of anesthesia on the ECG readings was not considered. Pregnant mice were anesthetized with a mixture of ketamine and xylazine, which is one of the most commonly used anesthetics in animal studies. Isoflurane was used in addition to ketamine/xylazine for anesthesia maintenance. In postnatal experiments, mice were anesthetized with isoflurane. It was reported that ketamine passes through the placental barrier and affects the fetus^58,59^. Ketamine/xylazine affect HR^60^, which is reduced by isoflurane in mice^61^. Therefore, anesthesia may represent a non-negligible factor in the results of this study, although its effect on fetal HR is unknown.

Second, sex differences regarding autonomic activities in the fetal and neonatal periods were not considered in this study. Because sex differences in symptoms in patients with ASD and VPA mice have been reported^33,34,54,55^, the validation of sex differences in the fetal and neonatal periods could be a very important and informative topic. Unfortunately, technical limitations did not allow the performance of ECG measurements using the same protocol from the fetal to the adult stages, which resulted in separate analyses for the fetal, neonatal, and adolescent/adult stages.

In conclusion, dysfunction and/or delay in the development of the autonomic nervous system can be a characteristic of prenatal VPA-treated ASD model mice. Although several issues remain to be resolved (such as the consideration of sex differences), because the activity of the autonomic nervous system can be assessed by ECG and human fetal ECG is feasible^29–31^, fetal and neonatal ECG measurements might be useful for the establishment of new diagnostic methods for ASD from an early developmental stage in the future studies.

## Materials and methods

### Animals

All handling and experimental procedures were performed in accordance with the Guidelines for the Care and Laboratory Animals of Tohoku University Graduate School of Medicine, and were approved by the Committee on Animal Experiments in Tohoku University (Sendai, Japan). The study's approval number is 2017MdA-334.

C57BL6/J mice (CLEA, Japan) were used in all studies. All mice were housed socially (3–5 mice in the same cage) in same-sex groups in a temperature-controlled environment under a 12 h/12 h light/dark cycle (lights on at 08h00, lights off at 20h00), with food and water available ad libitum*.*

### Prenatal VPA treatment

Female mice (7–19 weeks of age) were mated with age-matched male mice in the evening and checked for the presence of a vaginal plug on the next morning. We considered this day as embryonic day 0.5 (E0.5). On E12.5, 600 mg/kg of valproic acid sodium salt (VPA; Sigma, St. Louis, MO, USA) dissolved in saline solution (VPA mice) or saline solution alone (as a control; Sal mice) was injected into the subcutaneous fat of the neck of pregnant mice^7,9,33,62,63^. A 3-chambered social-approach test was performed using male mice to confirm whether VPA mice were an appropriate model of ASD (Sal: n = 8 from 3 mothers; VPA: n = 10 from 4 mothers). We collected and analyzed fetal ECG data at the E15.5 (Sal: n = 14 from 8 pregnant mice; VPA: n = 12 from 7 pregnant mice). Subsequently, ECG data were collected and analyzed at E18.5 (Sal: n = 9 from 6 pregnant mice; VPA: n = 10 from 6 pregnant mice) and on the postnatal day 0 (P0; Sal: n = 28 from 4 mothers; VPA: n = 19 from 3 mothers). The following data were excluded from further analysis (E15.5, Sal: n = 2 (arrhythmia); VPA, n = 2 (bradycardia and arrhythmia); E18.5, Sal: n = 3 (arrhythmia); VPA: n = 2 (bradycardia); P0, Sal: n = 1; outside the ± 3 SD range). Separate cohorts of experimentally naive pregnant female mice were used for each ECG experiment at E15.5, E18.5, and P0.

**Figure 6:**
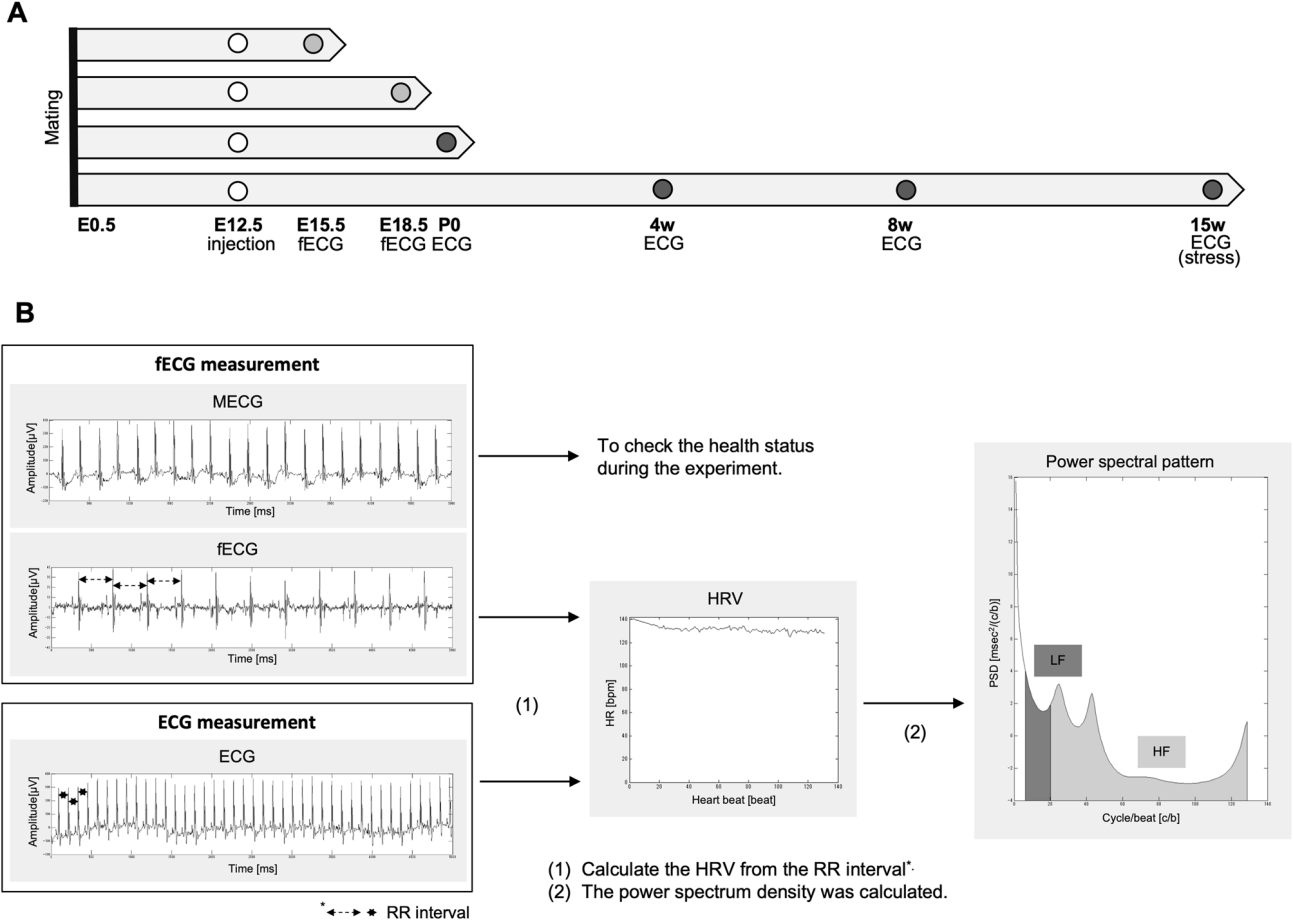
Experimental design of this study, and representative images and analytical procedure of maternal, fetal, and adult ECG. (**A**) Schedule of the experiments. (**B**) Representative images of maternal, fetal, and adult ECG measurements in mice. The power spectrum density was calculated from RR intervals. The low-frequency (LF) power indicates activities of both the sympathetic and parasympathetic nervous system, whereas the high-frequency (HF) power indicates parasympathetic nervous activity and the LF/HF ratio indicates sympathetic nervous activity.

In addition to the experiments described above, ECG data were collected and analyzed in mice born to mothers that were treated with VPA or saline, at 4 weeks of age (Sal: male, n = 16; female, n = 12, from 4 mothers. VPA: male, n = 9; female, n = 8, from 4 mothers) and at 8 weeks of age (Sal: male, n = 16; female, n = 12, from 4 mothers. VPA: male, n = 7; female, n = 7, from 4 mothers). In addition, ECG data were collected and analyzed in mice at 15 weeks of age (Sal: male, n = 15; female, n = 11, from 4 mothers. VPA: male, n = 6; female, n = 7, from 4 mothers) after 60 min of restraint stress and 15 min of restoration (Fig. 6A). In these experiments, ECG data were repeatedly collected at 4, 8, and 15 weeks of age in the same mice. Two male VPA mice died between 4 and 8 weeks of age, and one female VPA mouse had bradycardia at 8 weeks of age and died between 8 and 15 weeks of age. The following data were excluded from further analysis because of noise at the time of measurement (n = 1 male Sal mouse at 15 weeks of age, n = 1 female Sal mouse at 15 weeks of age, and n = 1 male VPA mouse at 15 weeks of age).

### Three-chambered social-approach test

The cohort of mice used in the 3-chambered social-approach test was independent from that used in the ECG experiments. We carried out the 3-chamber social-approach test according to our previous study^64^. The apparatus (51 × 34 × 22 cm) was divided into three chambers (17 × 34 cm) by transparent acrylic walls with rectangular openings (5 × 7 cm), thus allowing free movement of the test subjects between the chambers. The sides of the apparatus were made of black acrylic plastic, whereas the floor was made of white acrylic, to create a high contrast between the floor and the dark-colored mouse strain used in this study. Mice used as the novel mouse stimuli were age- and sex-matched C57BL6J mice. The stranger mice were habituated to the enclosure for 3 days prior to the test. A small stainless-steel holding cage (a wire cup (height, 10 cm; diameter, 8 cm) that could be inverted to keep the stimulus mouse in place) was used sequester the stimulus mouse in one chamber of the apparatus, allowing sniffing between mice but preventing full physical contact. During the sessions, a glass cup was placed on top of the holding cage to prevent the test subjects from climbing on top of it.

At the beginning of the test session, each test subject was allowed to habituate for 10 min to the apparatus with all of the compartments empty. The test mouse was then enclosed in the center compartment of the apparatus, and a novel stimulus mouse (stranger 1) was enclosed in a holding cage and placed in a side compartment. The location of stranger 1 alternated between the left and the right sides of the apparatus across test subjects. Following placement of stranger 1, the doors were reopened, and the test mouse was allowed to explore the entire apparatus for 10 min. Each mouse was again placed in the center compartment with the doors closed. A new stimulus mouse (stranger 2) was placed in the holding cage that had been empty during the previous session. The test mouse was once again allowed to explore all three compartments for a second 10 min session, to assess preference for social novelty. The time spent in each of the areas with the stranger mouse or empty cage was measured automatically using the ANY-Maze software (version 6.10, Stoelting Co., Wood Dale, IL).

### Fetal ECG measurements

Pregnant mice were anesthetized with subcutaneous ketamine (Ketalar 500 mg, 100 mg/kg; Daiichi-Sankyo) and xylazine (Rompun Inj Solution 2%, 10 mg/kg; Bayer) and maintained with inhalational isoflurane (0.5%, 260 ml/min; Forane AbbVie Inc.). The fetal ECG recording system for embryonic mice was as described in previous articles^32,65^. In short, three needle electrodes were attached to the pregnant mother, and two needle electrodes were inserted into the uterus, to attach them to the fetal chest and back. Fetal ECG recordings were collected simultaneously from two randomly selected fetuses from a pregnant mouse. ECG signals (sampling frequency, 1000 Hz) of pregnant mice and their fetuses were recorded for 15 min using a biomedical amplifier and recording system (Polymate AP1532; TEAC, Tokyo, Japan).

### Measurement of postnatal ECG

Mice were anesthetized with inhalational isoflurane (2%, 2 L/min; Forane AbbVie Inc.). Three needle electrodes were attached to the mice, and ECG signals (sampling frequency, 1000 Hz) were recorded from mice for 5 min using a biomedical amplifier and recording system (Polymate Mini; TEAC, Tokyo, Japan).

### Exposure to restraint stress

Animals were exposed to restraint stress by placing them in 50 mL plastic tubes with a few holes to maintain air flow for 60 min. After 15 min of restoration and 5 min of anesthesia using isoflurane, ECG was performed.

### Data analysis

ECG recording was performed for 15 min in the fetal stage and 5 min in the postnatal stage. For the analysis, 1 min from each stage was used. From the fetal stage data, 1 min between the 5th and 6th min was selected, and from the postnatal stage data, 1 min between the 1st and 2nd min was selected. As the mother's abdomen was opened for fetal ECG measurements and two needle electrodes were placed through the uterus, the first 5 min of recording, when the signal was not stable, were not used for data analysis. Conversely, for postnatal ECG measurements, three needle electrodes were attached to the mice and data from 1 min onward, when the signal was stable, were used for analysis. However, if the mentioned periods had noise and/or temporary arrhythmia, tachycardia, or bradycardia, the 1 min of data was selected from the 6th min and 10th min from the fetal stage data and from anywhere between the 1.5th min and 4th min from the postnatal stage data. The time slot that was selected from the fetal and postnatal stage data was the first 1 min period with a clear signal.

Beat-to-beat intervals were calculated from ECG data by finding the difference in time between two consecutive R-wave peaks. To assess the activities of autonomic nerves, we carried out a power spectrum analysis, as described previously^23^. The HRV was calculated from the RR interval of the fetal ECG or postnatal ECG data of each subject. The power spectral density was also calculated based on the pseudospectrum using the eigenvector method (peig), and the area of LF (7–20 cycle/beat) and HF (21-end cycle/beat) were calculated using the MATLAB software (version 2008b, MathWorks, USA). To analyze the data easily, LF and HF data were transformed to natural logarithm and defined as LF (ln) and HF (ln), respectively.

In this study, we considered LF (ln) as an indicator of the activity of the sympathetic and parasympathetic nerve, HF (ln) as an indicator of the activity of the parasympathetic nerve, and the LF (ln)/HF (ln) ratio as an indicator of the sympathetic nerve activity (Fig. 6B). We also analyzed the HR and short-term variability (STV). STV is another indicator of the parasympathetic nerve activity^47^. In this study, data that were outside the ± 3 SD range were excluded from further analysis. Noisy data, arrhythmia data, and bradycardia data were also excluded from further analysis. The fetal (E15.5 and E18.5) and P0 data were analyzed regardless of sex. Data pertaining to 4, 8, and 15 weeks of age were analyzed using sex as a between-subject factor.

### Statistical analysis

Data from the three-chambered social-approach test were analyzed using paired *t*-tests. The mean and standard error of the mean of the data were calculated. The ECG data were divided into the fetal (E15.5-E18.5), neonatal (P0), postnatal (4–8 weeks of age), and stress-loaded (15 weeks of age) stages of development for analysis, as the measurement methods and experimental protocols differed between fetal, neonatal, and subsequent developmental stages. Fetal ECG data of E15.5 and E18.5 mice were analyzed using two-way ANOVA with the between-subject factors of developmental stage (E15.5 vs. E18.5) and treatment (Sal vs. VPA). Significant ANOVA results were followed by Bonferroni post hoc comparisons, where applicable. ECG data of P0 mice were analyzed using one-way ANOVA with the between-subject factor of treatment (Sal vs. VPA). ECG data of 4-weeks- and 8-week-old mice were analyzed using three-way repeated measures ANOVA with the between-subject factors of sex (male vs. female) and treatment (Sal vs. VPA), and the within-subject factor of age (4 weeks vs. 8 weeks of age). For this analysis we used data from mice with complete data at both 4 and 8 weeks of age. Significant ANOVA results were followed by Bonferroni post hoc comparisons. ECG data of 15-weeks-old mice under the stress condition were analyzed using two-way ANOVA with the between-subject factors of sex (male vs. female) and treatment (Sal vs. VPA). Significant ANOVA results were followed by Bonferroni post hoc comparisons. All alpha levels were set at 0.05. We analyzed all data using the SPSS software (version 21, IBM, USA).

## Data Availability

The datasets generated during and/or analyzed during the current study are available from the corresponding author on reasonable request.

## References

[1] Miles JH (2011). Autism spectrum disorders–a genetics review. Genet Med..

[2] Zablotsky, B., Black, L. I. & Blumberg, S. J. Estimated prevalence of children with diagnosed developmental disabilities in the United States, 2014–2016. *NCHS Data Brief*, 1–8 (2017). 29235982

[3] Maenner MJ (2020). Prevalence of autism spectrum disorder among children aged 8 years - autism and developmental disabilities monitoring network, 11 sites, United States, 2016. MMWR Surveill. Summ..

[4] McPheeters ML (2011). A systematic review of medical treatments for children with autism spectrum disorders. Pediatrics.

[5] Dawson G (2010). Randomized, controlled trial of an intervention for toddlers with autism: the early start Denver model. Pediatrics.

[6] Ornoy A, Weinstein-Fudim L, Ergaz Z (2015). Prenatal factors associated with autism spectrum disorder (ASD). Reprod. Toxicol..

[7] Roullet FI, Lai JK, Foster JA (2013). In utero exposure to valproic acid and autism–a current review of clinical and animal studies. Neurotoxicol. Teratol..

[8] Ornoy A (2009). Valproic acid in pregnancy: how much are we endangering the embryo and fetus?. Reprod. Toxicol..

[9] Moldrich RX (2013). Inhibition of histone deacetylase in utero causes sociability deficits in postnatal mice. Behav. Brain Res..

[10] Tran NQV, Miyake K (2017). Neurodevelopmental disorders and environmental toxicants: epigenetics as an underlying mechanism. Int. J. Genom..

[11] Lee PR, Brady DL, Shapiro RA, Dorsa DM, Koenig JI (2007). Prenatal stress generates deficits in rat social behavior: reversal by oxytocin. Brain Res..

[12] Patin V, Lordi B, Vincent A, Caston J (2005). Effects of prenatal stress on anxiety and social interactions in adult rats. Brain Res. Dev. Brain Res..

[13] Grimm VE, Frieder B (1987). The effects of mild maternal stress during pregnancy on the behavior of rat pups. Int. J. Neurosci..

[14] Smith BL, Wills G, Naylor D (1981). The effects of prenatal stress on rat offsprings' learning ability. J. Psychol..

[15] Weller A, Glaubman H, Yehuda S, Caspy T, Ben-Uria Y (1988). Acute and repeated gestational stress affect offspring learning and activity in rats. Physiol. Behav..

[16] Dickerson PA, Lally BE, Gunnel E, Birkle DL, Salm AK (2005). Early emergence of increased fearful behavior in prenatally stressed rats. Physiol. Behav..

[17] Morley-Fletcher, S. *et al.* Prenatal stress in rats predicts immobility behavior in the forced swim test. Effects of a chronic treatment with tianeptine. *Brain Res.* **989**, 246–251, doi:10.1016/s0006-8993(03)03293-1 (2003). 10.1016/s0006-8993(03)03293-1 14556947

[18] Poltyrev T, Gorodetsky E, Bejar C, Schorer-Apelbaum D, Weinstock M (2005). Effect of chronic treatment with ladostigil (TV-3326) on anxiogenic and depressive-like behaviour and on activity of the hypothalamic-pituitary-adrenal axis in male and female prenatally stressed rats. Psychopharmacology.

[19] Beauchaine TP (2012). Physiological markers of emotional and behavioral dysregulation in externalizing psychopathology. Monogr. Soc. Res. Child Dev..

[20] Beauchaine TP (2015). Future directions in emotion dysregulation and youth psychopathology. J. Clin. Child Adolesc. Psychol..

[21] Beauchaine TP (2015). Respiratory sinus arrhythmia: a transdiagnostic biomarker of emotion dysregulation and psychopathology. Curr. Opin. Psychol..

[22] Wehrwein EA, Orer HS, Barman SM (2016). Overview of the anatomy, physiology, and pharmacology of the autonomic nervous system. Compr. Physiol..

[23] Kimura Y (1996). Power spectral analysis for autonomic influences in heart rate and blood pressure variability in fetal lambs. Am. J. Physiol..

[24] Montano N (1994). Power spectrum analysis of heart rate variability to assess the changes in sympathovagal balance during graded orthostatic tilt. Circulation.

[25] Harder R (2016). Heart rate variability during sleep in children with autism spectrum disorder. Clin. Auton Res..

[26] Tessier MP, Pennestri MH, Godbout R (2018). Heart rate variability of typically developing and autistic children and adults before, during and after sleep. Int. J. Psychophysiol..

[27] Thapa R (2019). Reduced heart rate variability in adults with autism spectrum disorder. Autism Res..

[28] Fenning RM (2019). Sympathetic-parasympathetic interaction and externalizing problems in children with autism spectrum disorder. Autism Res..

[29] Sato N (2011). Successful detection of the fetal electrocardiogram waveform changes during various states of singletons. Tohoku J. Exp. Med..

[30] Velayo CL, Funamoto K, Silao JNI, Kimura Y, Nicolaides K (2017). Evaluation of abdominal fetal electrocardiography in early intrauterine growth restriction. Front. Physiol..

[31] Oshio S (2018). A comparison study on safety and efficacy of maternal abdominal-lead fetal ecg under regulatory science. Adv. Clin. Transl. Res..

[32] Minato T (2018). Relationship between short term variability (STV) and onset of cerebral hemorrhage at ischemia-reperfusion load in fetal growth restricted (FGR) Mice. Front Physiol..

[33] Kazlauskas N, Seiffe A, Campolongo M, Zappala C, Depino AM (2019). Sex-specific effects of prenatal valproic acid exposure on sociability and neuroinflammation: relevance for susceptibility and resilience in autism. Psychoneuroendocrinology.

[34] Melancia F (2018). Sex-specific autistic endophenotypes induced by prenatal exposure to valproic acid involve anandamide signalling. Br. J. Pharmacol..

[35] Urraca N (2018). Significant transcriptional changes in 15q duplication but not Angelman syndrome deletion stem cell-derived neurons. Mol. Autism.

[36] Nicolini C, Fahnestock M (2018). The valproic acid-induced rodent model of autism. Exp. Neurol..

[37] Vegh, A. M. D. *et al.* Part and parcel of the cardiac autonomic nerve system: unravelling its cellular building blocks during development. *J Cardiovasc Dev Dis* **3**, doi:10.3390/jcdd3030028 (2016). 10.3390/jcdd3030028 PMC5715672 29367572

[38] Kataoka S (2013). Autism-like behaviours with transient histone hyperacetylation in mice treated prenatally with valproic acid. Int. J. Neuropsychopharmacol..

[39] Kawanai T (2016). Prenatal exposure to histone deacetylase inhibitors affects gene expression of autism-related molecules and delays neuronal maturation. Neurochem. Res..

[40] Yang EJ, Ahn S, Lee K, Mahmood U, Kim HS (2016). Early behavioral abnormalities and perinatal alterations of pten/akt pathway in valproic acid autism model mice. PLoS ONE.

[41] Kwon CH (2006). Pten regulates neuronal arborization and social interaction in mice. Neuron.

[42] Lugo JN (2014). Deletion of PTEN produces autism-like behavioral deficits and alterations in synaptic proteins. Front Mol. Neurosci..

[43] Segar JL, Mazursky JE, Robillard JE (1994). Changes in ovine renal sympathetic nerve activity and baroreflex function at birth. Am. J. Physiol..

[44] Oliveira V (2019). Early postnatal heart rate variability in healthy newborn infants. Front. Physiol..

[45] Graves BW, Haley MM (2013). Newborn transition. J. Midwifery Womens Health.

[46] Doherty, T. M., Hu, A. & Salik, I. Physiology, Neonatal. *StatPearls [Internet] *https://www.ncbi.nlm.nih.gov/pmc/articles/PMC7255313/ (2020). 30969662

[47] van Ravenswaaij-Arts, C. M., Kollee, L. A., Hopman, J. C., Stoelinga, G. B. & van Geijn, H. P. Heart rate variability. *Ann. Intern. Med.* **118**, 436–447, doi:10.7326/0003-4819-118-6-199303150-00008 (1993). 10.7326/0003-4819-118-6-199303150-00008 8439119

[48] Bharath, R., Moodithaya, S. S., Bhat, S. U., Mirajkar, A. M. & Shetty, S. B. Comparison of physiological and biochemical autonomic indices in children with and without autism spectrum disorders. *Medicina (Kaunas)* **55**, doi:10.3390/medicina55070346 (2019). 10.3390/medicina55070346 PMC6681286 31284658

[49] Koyama R, Ikegaya Y (2015). Microglia in the pathogenesis of autism spectrum disorders. Neurosci. Res..

[50] Presumey J, Bialas AR, Carroll MC (2017). Complement system in neural synapse elimination in development and disease. Adv. Immunol..

[51] Kazlauskas N, Campolongo M, Lucchina L, Zappala C, Depino AM (2016). Postnatal behavioral and inflammatory alterations in female pups prenatally exposed to valproic acid. Psychoneuroendocrinology.

[52] Bronzuoli MR (2018). Neuroglia in the autistic brain: evidence from a preclinical model. Mol. Autism.

[53] Rao M, Gershon MD (2016). The bowel and beyond: the enteric nervous system in neurological disorders. Nat. Rev. Gastroenterol. Hepatol..

[54] Green RM, Travers AM, Howe Y, McDougle CJ (2019). Women and autism spectrum disorder: diagnosis and implications for treatment of adolescents and adults. Curr. Psychiatry Rep..

[55] Pothineni NV, Shirazi LF, Mehta JL (2016). Gender differences in autonomic control of the cardiovascular system. Curr. Pharm. Des..

[56] Liu L, Zhang D, Rodzinka-Pasko JK, Li YM (2016). Environmental risk factors for autism spectrum disorders. Nervenarzt.

[57] Charkoudian N, Hart ECJ, Barnes JN, Joyner MJ (2017). Autonomic control of body temperature and blood pressure: influences of female sex hormones. Clin. Auton. Res..

[58] Li X (2018). Administration of ketamine causes autophagy and apoptosis in the rat fetal hippocampus and in PC12 cells. Front. Cell Neurosci..

[59] Zhao T (2014). Ketamine administered to pregnant rats in the second trimester causes long-lasting behavioral disorders in offspring. Neurobiol. Dis..

[60] Svorc P, Bacova I, Svorc P, Buzga M (2013). Autonomic nervous system under ketamine/ xylazine and pentobarbital anaesthesia in a Wistar rat model: a chronobiological view. Prague Med. Rep..

[61] Tan TP (2003). Assessment of cardiac function by echocardiography in conscious and anesthetized mice: importance of the autonomic nervous system and disease state. J. Cardiovasc. Pharmacol..

[62] Lucchina L, Depino AM (2014). Altered peripheral and central inflammatory responses in a mouse model of autism. Autism Res..

[63] Kotajima-Murakami H (2019). Effects of rapamycin on social interaction deficits and gene expression in mice exposed to valproic acid in utero. Mol. Brain.

[64] Sakakibara Y (2014). Developmental alterations in anxiety and cognitive behavior in serotonin transporter mutant mice. Psychopharmacology.

[65] Khandoker AH (2018). Regulation of maternal-fetal heart rates and coupling in mice fetuses. Conf. Proc. IEEE Eng. Med. Biol. Soc..

